# Artificial flexible sperm-like nanorobot based on self-assembly and its bidirectional propulsion in precessing magnetic fields

**DOI:** 10.1038/s41598-021-00902-6

**Published:** 2021-11-05

**Authors:** Nuoer Celi, De Gong, Jun Cai

**Affiliations:** 1 School of Mechanical Engineering and Automation, Beihang University, Beijing, 100191 China; 2 Shen Yuan Honors College, Beihang University, Beijing, 100191 China

**Keywords:** Biophysics, Engineering, Materials science, Nanoscience and technology, Physics

## Abstract

Sperm cells can move at a high speed in biofluids based on the flexible flagella, which inspire novel flagellar micro-/nanorobots to be designed. Despite progress in fabricating sperm-type robots at micro scale, mass fabrication of vivid sperm-like nanorobots with flagellar flexibility is still challenging. In this work, a facile and efficient strategy is proposed to produce flexible sperm-like nanorobots with self-assembled head-to-tail structure, and its bidirectional propulsion property was studied in detail. The nanorobots were composed of a superparamagnetic head and a flexible Au/PPy flagellum, which were covalently linked via biotin-streptavidin bonding with a high yield. Under precessing magnetic fields, the head drove the flexible tail to rotate and generated undulatory bending waves propagating along the body. Bidirectional locomotion was investigated, and moving velocity as well as direction varied with the actuating conditions (field strength, frequency, direction) and the nanorobot’s structure (tail length). Effective flagellar propulsion was observed near the substrate and high velocities were attained to move back and forth without U-turn. Typical modelling based on elastohydrodynamics and undulatory wave propagation were utilized for propulsion analysis. This research presents novel artificial flexible sperm-like nanorobots with delicate self-assembled head-to-tail structures and remarkable bidirectional locomotion performances, indicating significant potentials for nanorobotic design and future biomedical application.

## Introduction

Micro-/nanorobots with excellent swimming capability in fluids have become highly attractive due to their great potentials in biomedical applications^1–5^. These untethered robots can generate effective propulsion in fluidic environment and access desired sites at small scale to achieve special tasks. To date, various types of micro-/nanorobots have been developed via taking biological inspiration from motile microorganisms^6–8^. For example, inspired by corkscrew propulsion of *E. coli* bacteria, several researches have presented helical micro-/nanorobots actuated by rotating magnetic fields^9–13^. In fact, undulating propulsion is an easy-to-think motion method which is commonly utilized in nature from fishes to monotrichate eukaryotes^14–17^. In this case, sperm cell is typical with flagellar self-propulsive capability, which can move forward in low Reynolds number biofluids through the flagellum-generated transverse waves^18^, and also provides us with possibilities to realize effective flagellar propulsion at micro-/nanoscale.

To date, many researches are focused on designing and fabricating sperm-based micro-/nanorobots. Among these researches, some are focused on direct utilization of living motile sperm’s driving force, which can be integrated with magnetic guidance to form biohybrid microswimmers^19–21^. However, environmental sensitivity and inconsistent activity of the sperm cells greatly limit the practical applications. The other is to use the sperm’s overall shape to synthesize biotemplated robots at microscale with magnetized elastic flagellar structures^22,23^, yet the structure was relatively large, and the uncontrollable fabrication also hinders homogeneity in size as well as propulsion capability.

Enlightened by periodic undulations of the sperms, artificial sperm-shaped micro-/nanorobots have also attracted much attention, aimed to achieve biomimetics in structure and function. Among these researches, some are focused on integrated synthesis based on micro lithography or electrospinning technique, to construct a composite structure containing a magnetic head with a flexible polymer tail^24–27^. However, the relatively large size and complex fabrication process greatly limit biomedical uses of such millimeter-sized microrobots. Thereon, sperm-shaped self-assembled nanorobots with modular designs stand out, which integrate a tiny head with a slender tail for flagellar construction, and can be treated as an optimal way for essential imitation of the sperms. For example, a Fe_3_O_4_ microsphere could be connected with rigid Ni nanorod for sperm-shaped configuration, yet the desired flexibility and functionalization was lacked; or be linked with repolymerized bacterial flagellum to achieve flagellar propulsion, yet great diversity existed compared with the sperm’s shape^28–32^. Despite these tremendous efforts, to the best of our knowledge, no research has realized flexible sperm-like nanorobots with assembled head-to-tail structures. Thus it is imperative to develop facile and reliable fabrication methods to construct artificial sperm-like nanorobots, and study the corresponding flagellar propulsion at low Reynolds numbers.

Here, we propose artificial flexible sperm-like nanorobot synthesized via a facile self-assembly strategy using Fe_3_O_4_ nanobeads and flexible polymer flagella for the first time, which can generate bidirectional flagellar propulsion under precessing magnetic fields. Template-assisted electrochemical deposition was used to synthesize Au/PPy nanowires, and the streptavidin modified tails could be bonded with biotinylated magnetic nanobeads to form the sperm-like nanorobots. The self-assembled nanorobot was actuated in viscous glycerin and exhibited effective locomotion in forward or backward directions. A series of actuation experiments were conducted to characterize flagellar propulsion of the nanorobots, and bidirectional locomotion velocity under precessing magnetic fields of diverse parameters (strength, frequency, precession angle and direction) were studied in detail. Under a magnetic field of 70 Gs, 40 Hz and precession angle at 30°, the nanorobot could be actuated forward to reach a high velocity at 4.86 μm/s, and backward velocity at 3.17 μm/s could also be achieved when turning the precession axis around. Such vivid sperm-like nanorobot with flexible propulsion performance under magnetic actuation provides possibilities for flagellar nanorobot design and fabrication, and also indicates significant potentials for applications at low Reynolds numbers.

## Results and discussion

### Preparation and characterization of the sperm-like nanorobot

**Figure 1:**
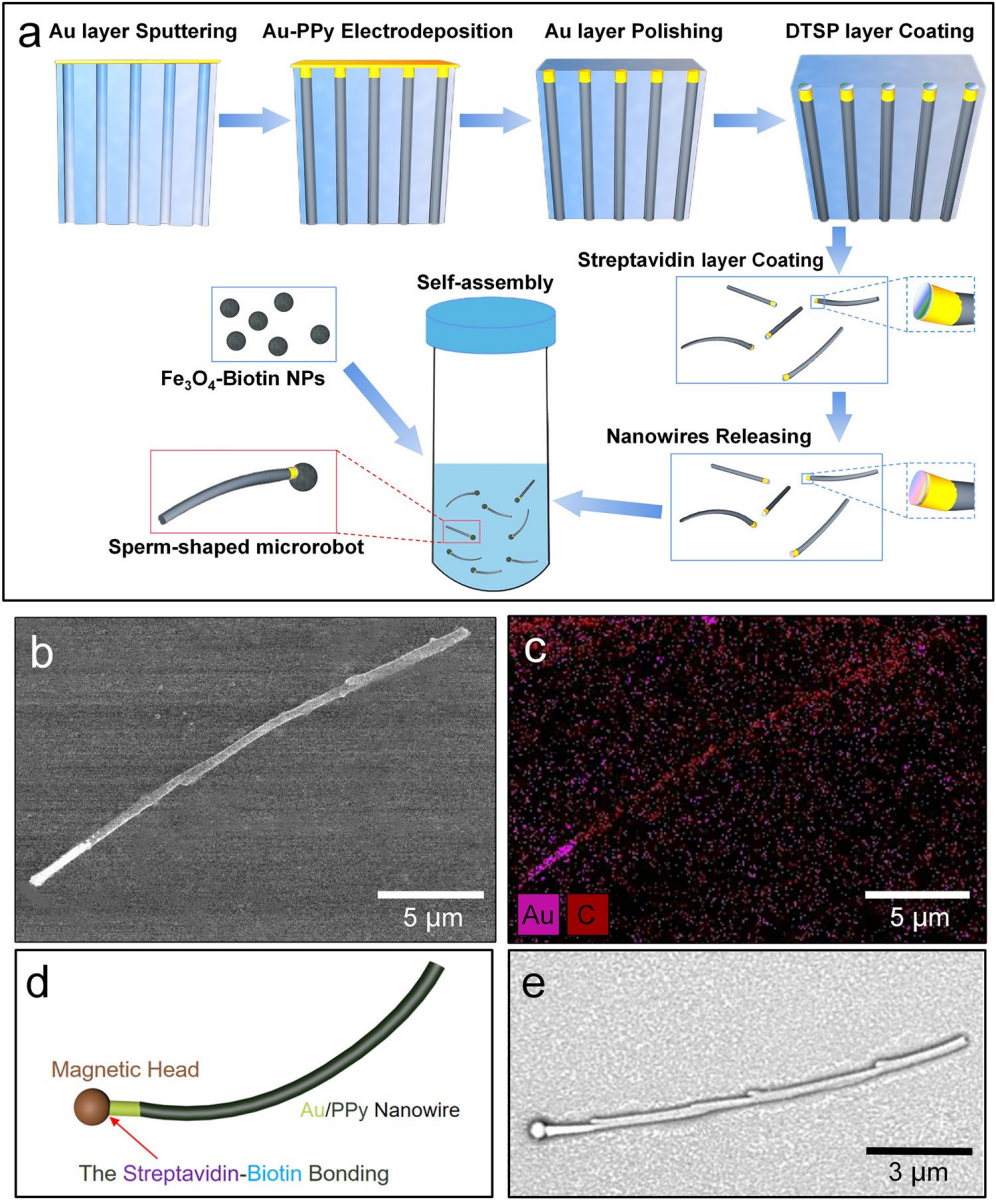
(**a**) Schematics of the fabrication process of the sperm-like nanorobots. (**b**,**c**) SEM image and EDS results of the as-prepared Au/PPy nanowire. (**d**,**e**) Structural schematics and SEM image of the self-assembled sperm-like nanorobot.

The fabrication process of sperm-like nanorobots was briefly shown in Fig. 1a. Firstly, we prepared flexible Au/PPy composite nanowires with the Au ends modified using streptavidin, which could act as artificial flagella of the nanorobots to generate undulatory wave propagation. Here, multi-step electrochemical deposition and monolayer modification were combined for fabrication. Au segments of 3 μm and PPy tails of given lengths were deposited in sequence. After mechanical polishing of the sacrificial gold layer, the Au tips of the nanowires were exposed and coated with monolayer of DTSP molecules, which could increase the contact area and improve the assembly efficiency for the following process. Then wet-etching of the Al_2_O_3_ templates was conducted and the nanowires were released to be modified with streptavidin. After that, the streptavidin-coated nanowires and biotin-coated Fe_3_O_4_ nanoparticles were mixed and vortexed simply at a speed of 150 r/min for 30 min. The biotin-coated Fe_3_O_4_ nanoparticles demonstrated superparamagnetic property without remanence and coercivity, and the VSM measurement was shown in Fig. S1. Thus the magnetization axis of the heads could keep consistent with the external magnetic field. During the assembly, spontaneous contact occurred between them and random self-assembly based on covalent bonds was achieved to form the sperm-shaped nanorobots. Ideally, the head and flagella are assembled as end-to-end. However, part of the heads and flagella assemble laterally with a minor offset (Fig. S2). This was ascribed to the brittle surface of the AAO templates, which was easy to generate micro cracks in the polishing process and lead the reaction solution to infiltrate. Thus the DTSP and streptavidin layers were not only coated on the end surfaces, but also penetrate slightly to modify the sidewalls close to the ends, resulting in the lateral assembly. For magnetic actuation, this offset is negligible since the integral sperm-shaped assembly structure as well as the overall locomotion are not affected.

Morphology and elemental analysis of the artificial flagella were conducted, and the SEM and EDS results were shown in Fig. 1b,c. Distinct two-stage structure can be observed on the nanowire, which corresponds to the Au tip and the PPy body, respectively. Flexibility of the artificial flagellum is presented with slightly bending in this view (Fig. S5). Schematics of the self-assembled nanorobot’s head-to-tail structure and the corresponding SEM image were shown in Fig. 1d,e, respectively. It can be observed that an intact self-assembled microstructure has been successfully achieved between the magnetic head and the flexible flagellum, to form the sperm-like nanorobot. The detailed assembly structure was shown in Fig. S3a, and the optical image of multiple assembled nanorobots was shown in Fig. S3b to demonstrate the high yield. Due to the scalable versatility of the biotin-modified Fe_3_O_4_ head and the ppy flagellum, the nanorobot shows good potentials for further functionalization towards biomedical uses.

### Theoretical analysis of the nanorobot’s propulsion

The nanorobot in this study is fabricated via self-assembly using a superparamagnetic nanobead and an artificial flexible ultra-thin nanorod. Assuming the head has a magnetic moment *M* in the direction under the magnetic field, the flexible flagellum has a body length *L* with a given elasticity modulus. For an arbitrary point *P* along the tail, we mark it with a generalized coordinate *q* respective to the head and describe its position at time *t* with vector *p(q, t)*, which can also be transferred to the head’s frame as

1 $$ p\left( {q,t} \right) = \left( {q\quad \phi_{y} \left( {q,t} \right)\quad \phi_{z} \left( {q,t} \right)} \right) $$

where *ϕ*_*y*_*(q,t)* and *ϕ*_*z*_*(q,t)* represent the corresponding deformation along Y and Z axis, respectively.

Then the moving Frenet–Serret frame can be established on point *P* along local tangent, normal, and bi-normal directions:

2 $$ b = \frac{{dp\left( {q,t} \right)/dq}}{{\left| {\left| {dp\left( {q,t} \right)/dq} \right|} \right|}},\quad a = \frac{db/dq}{{\left| {\left| {db/dq} \right|} \right|}},\quad c = a \times b $$

The head can orient itself to the time-varying magnetic field due to the magnetic torque exerted on the dipole. Due to the head’s rigid body rotation, bending waves are generated and can be propagating along the flexible flagellum. According to the typical resistive force theory, the force and torque balance based on magnetic and fluidic fields can be expressed as:

3 $$ \left[ {\begin{array}{*{20}c} {F_{m} + F_{d} } \\ {T_{m} + T_{d} } \\ \end{array} } \right] = 0 $$

Here, gravity is neglected, *F*_*m*_ and *T*_*m*_ are magnetic force and torque that can be expressed as:

4 $$ \left[ {\begin{array}{*{20}c} {F_{m} } \\ {T_{m} } \\ \end{array} } \right] = \left[ {\begin{array}{*{20}c} {V\left( {M \cdot \nabla } \right)B} \\ {VM \times B} \\ \end{array} } \right] $$

*F*_*d*_ and *T*_*d*_ are fluidic drag force and torque, which can be decomposed into contributions of the head and the tail:

5 $$ \left[ {\begin{array}{*{20}c} {F_{{d,{ }head}} } \\ {T_{{d,{ }head}} } \\ \end{array} } \right] = \left[ {\begin{array}{*{20}c} {D_{h} } & { - D_{h} S_{h} } \\ {S_{h} D_{h} } & {N_{h} } \\ \end{array} } \right]\left[ {\begin{array}{*{20}c} V \\ \Omega \\ \end{array} } \right] $$

6 $$ \left[ {\begin{array}{*{20}c} {F_{{d,{ }tail}} } \\ {T_{{d,{ }tail}} } \\ \end{array} } \right] = \mathop \int \limits_{0}^{L} \left[ {\begin{array}{*{20}c} {RCR^{T} } & { - RCR^{T} S_{t} } \\ {S_{t} RCR^{T} } & { - S_{t} RCR^{T} S_{t} } \\ \end{array} } \right]\;dl\left[ {\begin{array}{*{20}c} V \\ \Omega \\ \end{array} } \right] $$

where *V* and *Ω* are propulsion and rotation velocity matrices, *D*_*h*_ and *N*_*h*_ are resistance matrices for the head, and *C* represents the resistance coefficient matrix for the tail. Besides, *R* is the rotation matrix between the local Frenet-Serret coordinates and the nanorobot’s frame, *S*_*h*_ and *S*_*t*_ are the corresponding position transformation matrices for the head and the tail.

The above equations describe the elastohydrodynamics of the sperm-shaped nanorobot swimming in viscous fluids^27^. In the experiments, the nanorobot was actuated to swim in the horizontal plane (XY) and was investigated from the top view. Apart form the elastohydrodynamics, a simplified undulatory propagation modelling could also be used^33,34^. Swimming of the nanorobot in the X direction can be simplified into two orthogonal components in the XY and XZ planes. Motion of the nanorobot in each plane can be treated as a bending wave propagation across the two distal ends, and relative phase lag between the head and tail in two planes are noted as *ϕ*_*xy*_ and *ϕ*_*xz*_. Here, flagellar propulsion of the sperm-like nanorobot along X direction can be ascribed to the overall oscillation along the body induced by two separate oscillations in both XY and XZ planes as:

7 $$ y\left( {x,t} \right) = a_{1} \sin (2\pi ft)\left( \frac{x}{L} \right) + a_{2} \sin (2\pi ft + \varphi_{xy} )\left( \frac{x}{L} \right)^{m} $$

8 $$ z\left( {x,t} \right) = a_{3} \sin (2\pi ft)\left( \frac{x}{L} \right) + a_{4} \sin (2\pi ft + \varphi_{xz} )\left( \frac{x}{L} \right)^{m} $$

where *a*_1_ and *a*_3_ are amplitudes of magnetic oscillation that correspond to the head, *a*_2_ and *a*_4_ are amplitudes of fluidic oscillation that correspond to the tail. Besides, *f* is the actuation frequency, and *m* is the curvature of bending deformation.

The propulsive forces in two planes can be expressed as

9 $$ F_{x} \left( {xy} \right) = \mathop \int \limits_{0}^{L} C_{\parallel } \left[ {\left( {\frac{{C_{ \bot } }}{{C_{\parallel } }} - 1} \right)\frac{dy}{{dt}}\frac{dy}{{dx}} - V_{x} \left( {xy} \right)} \right]dl $$

10 $$ F_{x} \left( {xz} \right) = \mathop \int \limits_{0}^{L} C_{\parallel } \left[ {\left( {\frac{{C_{ \bot } }}{{C_{\parallel } }} - 1} \right)\frac{dz}{{dt}}\frac{dz}{{dx}} - V_{x} \left( {xz} \right)} \right]dl $$

where *dl* is an infinitesimal section along the body, $C_{\parallel }$ and $C_{ \bot }$ are the drag coefficients in the tangential and normal directions. Besides, $V_{x} \left( {xy} \right)$ and $V_{x} \left( {xz} \right)$ are two contributions of the X-direction velocity resulted from oscillations in XY and XZ planes, respectively. When the nanorobot reaches a steady swimming state, the total force equals zero, and the velocity components can be calculated to be

11 $$ V_{x} \left( {xy} \right) = \pi \left( {\frac{{C_{ \bot } }}{{C_{\parallel } }} - 1} \right)\left( {\frac{m - 1}{{m + 1}}} \right)\frac{{a_{1} a_{2} }}{Lf}\sin \left( {\varphi_{xy} } \right) $$

12 $$ V_{x} \left( {xz} \right) = \pi \left( {\frac{{C_{ \bot } }}{{C_{\parallel } }} - 1} \right)\left( {\frac{m - 1}{{m + 1}}} \right)\frac{{a_{3} a_{4} }}{Lf}\sin \left( {\varphi_{xz} } \right) $$

And the resultant velocity along X-direction can be expressed as

13 $$ V_{x} = V_{x} \left( {xy} \right) + V_{x} \left( {xz} \right) $$

Hence, we can deduce that a phase difference in oscillation is requisite to generate effective locomotion with a nonzero velocity. The oscillations in two perpendicular planes undergo different fluidic drag conditions due to the substrate-induced asymmetry, and diverse amplitudes as well as phase lags are stimulated. The resultant motion of the nanorobot is determined by combination of the decomposed oscillations, which can result in a balance between propulsion and retarding in the form of forward or backward locomotion. In this case, dynamic magnetic field with a precessing angle is essential for swimming direction reversal since spatial oscillations become accessible to lead to bidirectional resultant.

### Propulsion performance of the sperm-like nanorobot

To actuate the sperm-like nanorobot and test its propulsion performance, a precessing magnetic field or a so-called conical rotating magnetic field was applied using a custom-made triaxial Helmholtz coil system. The externally actuated magnetic field can be expressed as

14 $$ \vec{B} = B_{yz} \left[ {\cos (2\pi ft} \right)\overrightarrow {{e_{y} }} + \sin (2\pi ft)\overrightarrow {{e_{z} }} ] + B_{x} \overrightarrow {{e_{x} }} $$

where *B*_*yz*_ and *f* are the amplitude and rotating frequency of the circular polarized component of the magnetic field. And *B*_*x*_ is the static component of the magnetic field. Besides, $\overrightarrow {{e_{x} }}$, $\overrightarrow {{e_{y} }}$, $\overrightarrow {{e_{z} }}$ are the unit vectors along the corresponding coordinate axis.

**Figure 2:**
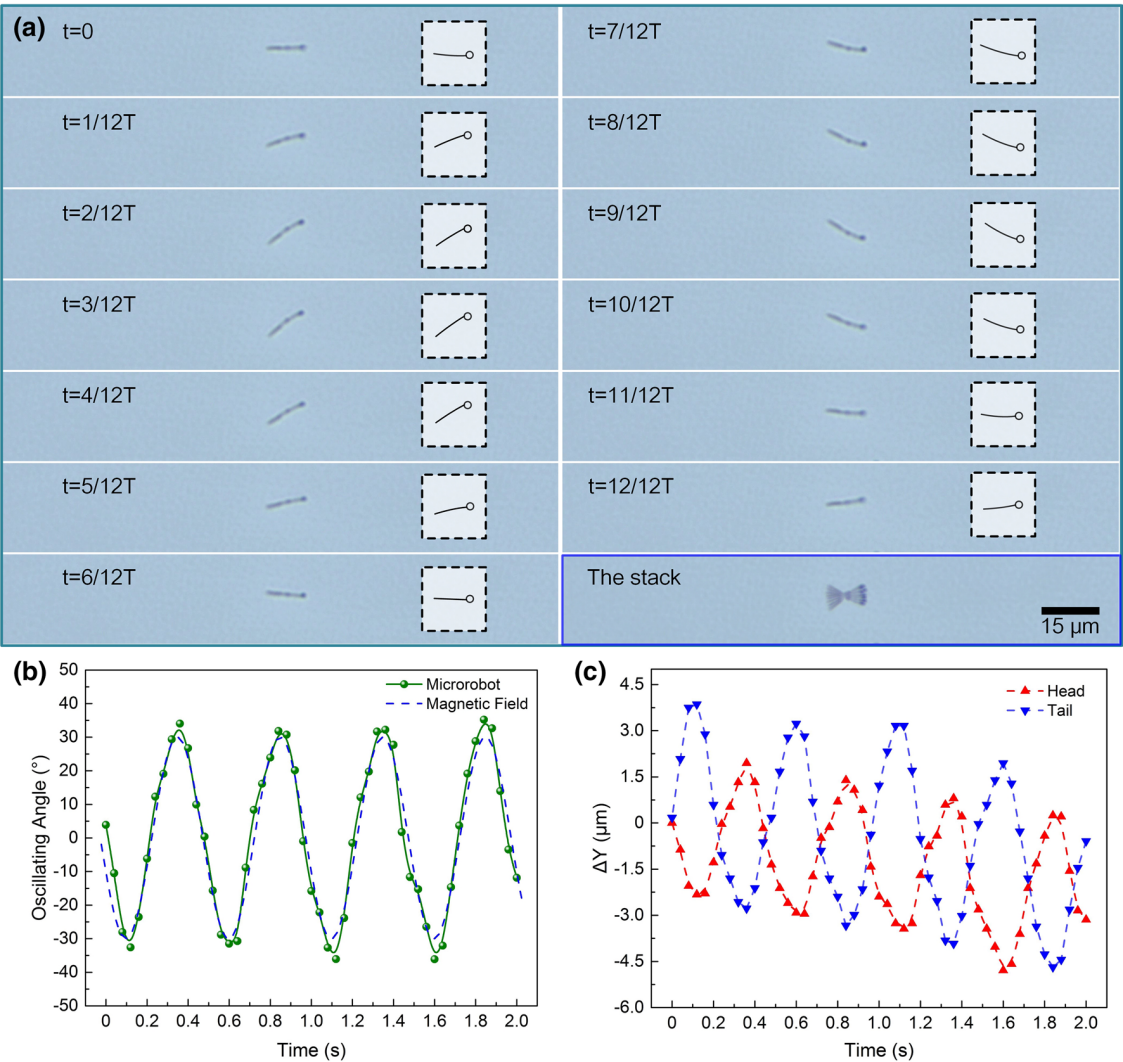
(**a**) Time-lapse images of a sperm-like nanorobot’s flagellar oscillation over one propulsion period (T = 0.5 s) actuated under externally applied precessing magnetic field (B = 100 Gs, f = 2 Hz, θ = 30°). The insets were morphing postures of the nanorobot at corresponding stages, and the stack image was also shown to demonstrate the whole oscillation process under magnetic actuation. (**b**) The plot describes the nanorobot’s oscillation angles observed in the two-dimensional view under continuous magnetic actuation. The dashed blue line is the real-time oscillation angle of the applied precessing magnetic field. (**c**) The position displacement of the nanorobot’s head and tail tip in the oscillation process under magnetic actuation.

The resultant field vector at the head of the nanorobot was rotating in a cone-like path, and the angle between the field vector and the cone axis was noted as the precessing angle (*θ*) of the actuation field. For flagellar propulsion of sperm-like nanorobots, undulatory motion theory has been developed as described above, which is focused on the bending wave propagating process along the body. Actuated by the magnetic torque exerted on the head, continuous rotation is triggered to drive the body to fluctuate. The stimulated wave propagates along the flagellum yet different oscillating amplitudes are generated from the head to the tail. Flexibility of the artificial flagellum contributes to effective undulatory propagation and a constant phase lag exists compared to the magnetic head. Here, time-dependent deformation of the flexible flagellum was measured over one whole period and morphing changing process of the body was also illustrated. The nanorobot was actuated to oscillate by a 2 Hz precessing field (*B* = 100 Gs, *θ* = 30°) (Movie 1). The results indicate the whole propagating process of the generated wave during helical flagellar propulsion, and a distinct oscillating angle can be observed from the superimposed image as shown in Fig. 2a. Such nanorobot could be actuated to precess synchronously with the low-frequency alternating magnetic field.

A planar coordinate system was defined to facilitate position recording and calculation. In this view, the upward tail tip of the nanorobot corresponds to a positive value of oscillating angle yet the downward tail tip corresponds to a negative one. As shown in Fig. 2b, we measured the oscillating angle change of the nanorobot in a given time period, which exhibited a sinusoidal pattern with time and kept consistent with the precessing field’s oscillation. To investigate the relative position relations between the nanorobot’s head and flagellum, we further recorded the displacements of the head and the tail tip along the Y axis, with respect to the original position (Y = 0) at t = 0 s. As shown in Fig. 2c, the displacement waveforms near the distal end as well as the head were recorded. Both the head and the flagellum undergo a sinusoidal waving locomotion in the direction perpendicular to the cone axis, and a fixed phase diversity exists between them. The head and the flagellum exhibit diverse oscillation amplitudes during propulsion, in which the tail tip oscillates with a larger amplitude compared with the head. In this case, the nanosphere head dominates the magnetic response and acts as an oscillation source. It is actuated to precess around and drives the flagellum to rotate in the same pattern with a phase lag under the sinusoidal alternating magnetic field. It also proves that asymmetrical shape deformation occurs to cater the scallop theorem and achieve effective locomotion as a result. At the same time, a negative positional offset of the swimmer’s body can be observed, which is related to the minor lateral drift during swimming.

### Bidirectional locomotion property of the nanorobot

**Figure 3:**
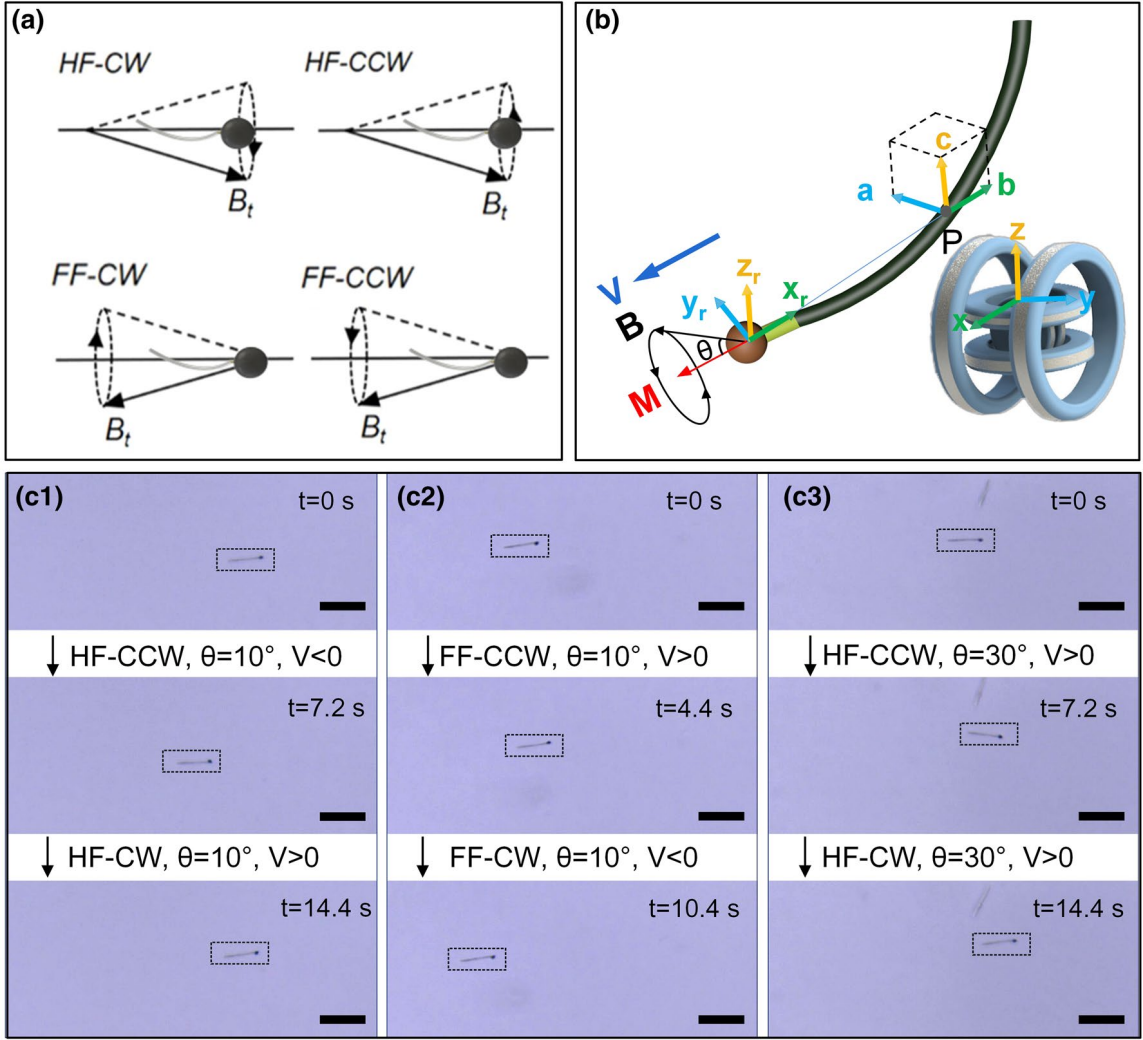
(**a**) Definition of various spatially oriented precessing magnetic fields with respect to the sperm-like nanorobot. (**b**) Schematics of a sperm-like nanorobot actuated by a precessing magnetic field in triaxial Helmholtz coils. (**c**)Time-lapse images of a nanorobot’s motion under magnetic fields of diverse precessing directions and angles, which were set at a constant strength (100 Gs) and frequency (40 Hz). The position of the nanorobot is marked with an enclosing dashed rectangle box. Scale bars: 15 μm.

For magnetic actuation of the sperm-like nanorobot, four types of spatially directional precessing magnetic fields could be defined according to the initial orientation of the nanorobot (Fig. 3a). When the field vector exhibited an acute angle with the flagellum-head direction, the field was defined as a “Head-pointing Field” (HF), and the rotating direction was further classified into counter-clockwise (CCW) or clockwise (CW) types on the basis of the right-hand rule. Schematics of the sperm-like nanorobot actuated by a HF type field with a precessing angle θ was shown in Fig. 3b. Similarly, when the field vector was pointing in the head-flagellum direction, it could be defined as a “Flagellum-pointing Field” (FF) with a CCW or CW rotating directions. Since the head was superparamagnetic, the diversity of fields could not cause orientation change, which was quite different from ferromagnetic nanorobots. In this case, ferromagnetic Ni/PPy nanowires were also driven by the four precessing fields to demonstrate such diverse effects (Movie 5 and 6). For Ni/PPy nanorobot with magnetic remanence, it could easily turn around for coincident magnetic alignment and synchronous precessing when the field axis (f = 2 Hz) was turned from HF to FF types. Yet in a high-frequency field (f = 5 Hz), when the field axis was abruptly turned, the Ni/PPy nanorobot failed to precess synchronously with the fields and could not turn around since the magnetic torque was insufficient to overcome the fluidic drag. According to previous researches, rotating direction (CW and CCW) of the fields does not affect locomotion direction of achiral Ni/PPy nanorobots, which have no back and forth motion behaviour^16,35^. However, for the superparamagnetic sperm-like nanorobot, all four types of fields could be used for effective actuation without U-turn, thus we continued to implement propulsion tests under four types of magnetic fields to fully verify the above views.

To study the locomotion directionality, diverse precessing angles and directions were adopted for experimental comparison (Movie 2). As shown in Fig. 3c, the field strength and frequency were set to be constant at 100 Gs, 40 Hz, yet the spatial directions were different. In Fig. 3c-1, when a field of HF-CCW at θ = 10° was applied, backward locomotion in the flagellum-pointing direction could be observed, which corresponded to a negative velocity (V < 0). However, when the field was set to be precessing in the CW direction, the nanorobot changed to swim forward on the contrary. In Fig. 3c-2, the magnetic field (θ = 10°) was tuned to be a FF type, the nanorobot kept the original orientation without turning around due to superparamagnetic property of the head. For magnetic field transformation, a HF-CW type field could be tuned to FF-CW type as just turned the precessing axis 180°, thus the rotating direction of the superparamagnetic head was reversed and the nanorobot could be driven to oscillate and move in an opposite direction. This is attributed to the chirality of the sperm-like nanorobot combined with the nonideal self-assembled structural configuration as well as the time-dependent undulatory dynamics. Therefore, field switching between HF and FF types or just between CW and CCW directions can realize bidirectional locomotion of the nanorobot without a U-turn trajectory, which is different from typical single-tailed magnetic micro-/nanoswimmers. The results also confirmed that HF-CCW and FF-CW fields could achieve the same effect to actuate the nanorobot, and the same relations existed between HF-CW and FF-CCW fields.

In addition, the influence of the actuation field’s precession angle was also investigated as shown in Fig. 3c-3. Under a HF type field at θ = 30°, the nanorobot could be effectively actuated forward in both CCW and CW directions, which was quite different with the results demonstrated in Fig. 3c-1. It could be explained that actuation field of a large precessing angle induced relatively violent precession with higher oscillation frequency, and the chirality was no longer valid in this case. The results confirmed that flexible switching between swimming forward and backward could be achieved via tuning the actuation field parameters, including precessing direction and angle as well as field frequency. In the future, such bidirectional propulsion property can be applied to control multiple sperm-like nanorobots with distinguished strategies and complete cooperative tasks.

**Figure 4:**
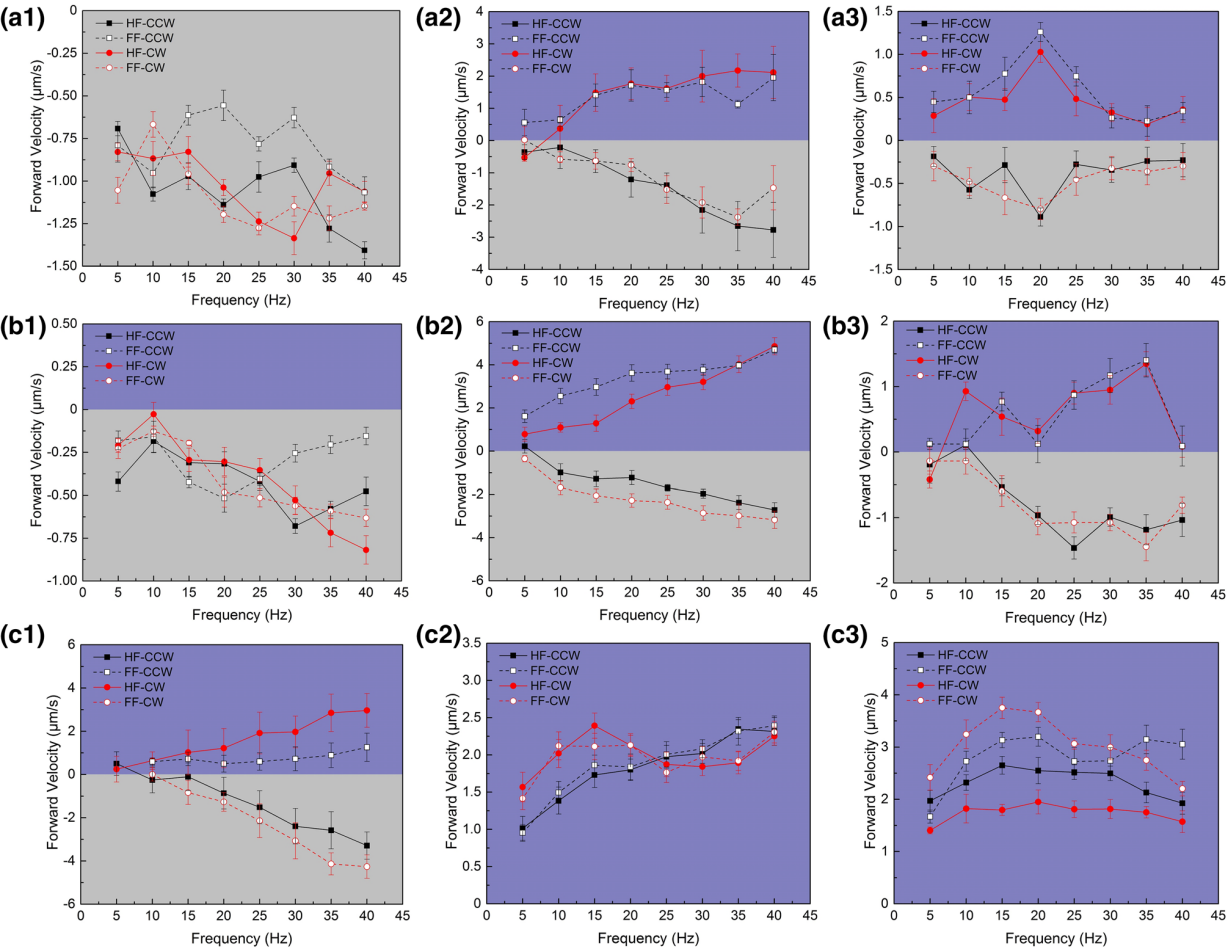
Locomotion velocity of the sperm-like nanorobots actuated under various types of precessing magnetic fields (HF-CCW, HF-CW, FF-CCW, FF-CW) with given parameters (field strength B, precession angle θ) over a frequency range (5–40 Hz). (**a1**–**a3**) B = 50 Gs, θ = 10°, 30°, 60°. (**b1**-**b3**) B = 70 Gs, θ = 10°, 30°, 60°. (**c1**-**c3**) B = 100 Gs, θ = 10°, 30°, 60°.

Based on the preliminary actuation study above, actuation experiments under fields of various parameters were conducted, and locomotion directions as well as resultant velocities were systematically measured. As shown in Fig. 4a-1 to 3, the field strength was set to be constant at 50 Gs, yet the precessing angles were 10°, 30°, 60°, respectively. Four types of fields (HF-CCW, HF-CW, FF-CCW, FF-CW) at a given frequency ranging from 5 to 40 Hz were applied to actuate the nanorobot. The positive velocity corresponded to swimming forward yet the negative velocity represented backward propulsion. At a lower value of precession angle (θ = 10°), the nanorobot was actuated to swim backward over the frequency range regardless of spatial directions of the fields. However, the results at higher precession angles (θ = 30° and 60°) were completely different since both forward and backward locomotion could be observed. Specifically, the nanorobot was actuated forward under HF-CW or FF-CCW fields, yet backward under HF-CCW or FF-CW fields. A highly effective forward velocity of 2.11 μm/s and backward velocity of 2.77 μm/s could be easily obtained at θ = 30° via tuning the precession directions of the field, indicating distinct bidirectional propulsion property which was different from previously reported sperm-shaped microswimmers.

As shown in Fig. 4b-1 to 3, under precessing magnetic fields of 70 Gs, the propulsion results were similar with the cases in 50 Gs when other parameters kept the same. The nanorobots still kept swimming backward at θ = 10°, yet locomotion differentiation occurred when θ = 30° or 60°. However, when the field strength was large enough (100 Gs), it turned to be much easier for the nanorobots to move forward. Specifically, both HF-CW and FF-CCW fields could successfully drive the nanorobots forward at θ = 10°, despite backward locomotion was still destined under HF-CCW or FF-CW fields. A large forward velocity of 2.96 μm/s and backward velocity of 4.26 μm/s could both be achieved at 40 Hz using the opposite precessing directions (HF-CW and FF-CW fields, respectively). As increasing precessing angle of the 100 Gs fields (θ = 30°, 60°), robust forward propulsion turned to be dominated regardless of the spatial directions of the actuation fields. In this case, a significantly high forward velocity could be obtained over a wide frequency range.

Thereon, we measured and summarized locomotion directions of the nanorobot actuated by precession magnetic fields of diverse parameters, and the results were listed in Table 1. From the observations in our experiments, it can be deduced that locomotion direction of the sperm-like nanorobot is determined by multiple actuation parameters including field strength, frequency, precessing angle as well as direction. In the experiments, the sperm-like nanorobots generally tended to move forward under precessing magnetic fields of HF-CW or FF-CCW types. To propel the nanorobots forward, precessing magnetic fields of an intensified strength as well as a large precessing angle were desired for actuation, which was also consistent with the velocity measurement results in Fig. 4.

**Table 1:** Locomotion directions of the sperm-like nanorobot actuated by a precessing magnetic field of given parameters over a frequency range (5–40 Hz). Here, “ + ” represents forward locomotion yet “−” represents backward locomotion.

θ	B (Gs)	HF-CW/HF-CCW	FF-CW/FF-CCW
10°	50	−/−	−/−
	70	−/−	−/−
	100	+ /−	−/ +
30°	50	+ /−	−/ +
	70	+ /−	−/ +
	100	+ / +	+ / +
60°	50	+ /−	−/ +
	70	+ /−	−/ +
	100	+ / +	+ / +

**Figure 5:**
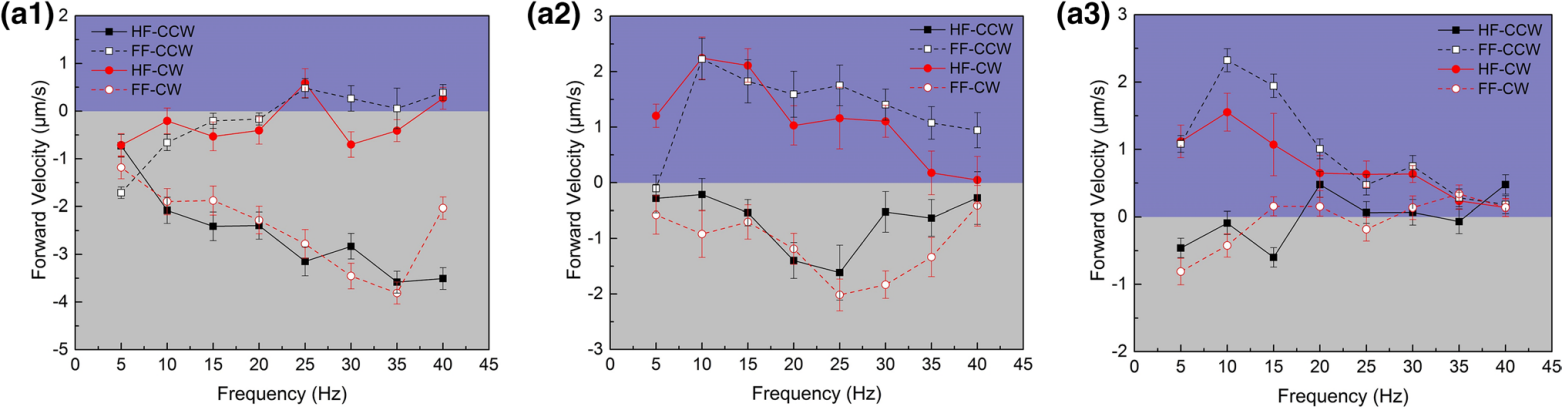
Locomotion velocity of the short-tailed nanorobot actuated under a 100 Gs precessing magnetic fields (HF-CCW, HF-CW, FF-CCW, FF-CW) over a frequency range (5–40 Hz) at given precession angles θ. (**a1**-**a3**) θ = 10°, 30°, 60°.

Moreover, we also fabricated nanorobots with shorter flagella, which were synthesized via controlling the electrochemical deposition time. In Fig. S4, SEM image of the short-tailed nanorobot (tail length about 8 μm) was demonstrated. Actuation experiments of such nanorobot under precessing magnetic fields of 100 Gs were carried out (Movie 3 and 4) and locomotion velocities were calculated as shown in Fig. 5. Similarly, four directional types of fields and precessing angles of 10°, 30°, 60° were applied successively for flagellar propulsion. Bidirectional locomotion could be distinctly observed under fields of any precession angles, which was quite different compared with the long-tailed nanorobots that tended to move forward under the same actuation condition. Under the 100 Gs magnetic field of a moderate precessing angle (θ = 30°), typical bidirectional property could be observed and relatively high locomotion velocity exceeded 2 μm/s could be achieved both in forward and backward directions. The nanorobot could be actuated to move forward under precessing magnetic fields of HF-CW or FF-CCW types, and move backward under HF-CCW or FF-CW fields, which were still similar with the experimental results of long-tailed ones actuated under 70 Gs fields. With diversity in body length, the sperm-like nanorobots exhibited different sensitivity to the dynamic fields yet the locomotion directions kept basicly consistent. It can also be predicted that short-tailed nanorobots tend to move forward under precessing fields of an enhanced intensity.

## Conclusion

In summary, we presented novel flexible sperm-like nanorobots based on a simple and facile preparation method. Based on tunable electrochemical deposition of flexible flagella and self-assembled bonding with magnetic nanobeads, the mass produced sperm-like nanorobots were endowed with flexible head-to-tail structures. The integrated nanorobot was actuated in glycerin solution and exhibited effective bidirectional locomotion properties with respect to the dynamic magnetic fields. Motion velocities under diverse magnetic fields were measured, showing the directions were mainly determined by the actuation parameters including field intensity, frequency as well as spatial directions. Under a precessing magnetic field of 70 Gs at θ = 40°, the nanorobot could be actuated forward to reach a high velocity at 4.86 μm/s (about 0.37 body length/s), and backward velocity of 3.17 μm/s (about 0.24 body length/s) could also be achieved when turning the field direction. Based on tuning the actuation fields, swimming forward and backward could be switched without U-turn, which was useful for flexible motion control in viscous fluids. This work demonstrated ultrasmall flexible sperm-like nanorobot with effective bidirectional propulsion under magnetic actuation, which provided significant exploration of reliable nanorobotic models at low Reynolds numbers. In future work, it is necessary to explore group manipulation and further biomedical applications of such nanorobots.

## Methods

### Reagents and materials

In the experiments, sodium hypophosphite (NaH_2_PO_2_·H_2_O), chloroauric acid (HAuCl_4_·4H_2_O), trisodium citrate (Na_3_C_6_H_5_O_7_·2H_2_O), citric acid (C_6_H_8_O_7_), pyrrole (C_4_H_5_N), boric acid (H_3_BO_3_), ferric chloride (FeCl_3_·6H_2_O), ferrous chloride (FeCl_2_·4H_2_O), sodium hydroxide (NaOH), glycerol (C_3_H_8_O_3_), dimethyl sulfoxide (DMSO), dithio-bis-succinimidyl propionate (DTSP), biotin and streptavidin solutions were all purchased from Lan Yi Chemical Reagent Company (Beijing, China). Biotin modified Fe_3_O_4_ nanoparticles (SVM-025-5H) were purchased from Spherotech.

### Synthesis of the flexible Au/PPy nanowires

The commercial AAO template with pore size of 200 nm was purchased from Whatman Anodisc. Firstly, 100 nm of gold was coated on one side of the template, acting as a conductive working electrode via magnetic sputtering technique. Then the AAO template was placed in a self-made electrodeposition container, and Ag/AgCl reference electrode as well as Pt wire electrode was used for subsequent deposition. The Au-coated membrane was immersed in the prepared gold plating solution (containing 30 g/L H_3_BO_3_ and 34 g/L HAuCl_4_·4H_2_O), and the Au section was deposited at a DC voltage of − 0.2 V. Next, deposition of PPy nanorods was conducted using prepared PPy plating solution (containing 19.25 g/L citric acid and 6.95 mL/L pyrrol) at +0.8 V. PPy flagella of various lengths could be synthesized via adjusting the deposition time. After that, mechanical polishing was conducted with 3–4 μm alumina powder to remove the sputtered sacrificial gold layer.

### Modification process of the nanowires

Firstly, the Au ends of the nanowires (retained in the AAO templates) were exposed to a DMSO solution containing DTSP (4 mM), and immersed for 6 hours at 25 °C to modify a self-assembled monolayer of DTSP on the end of gold segment. Then, streptavidin solution (0.5 mg/mL) was added to immerse the polished surface at room temperature for 6 hours, to covalently bond streptavidin to the DTSP modified Au ends. After streptavidin functionalization, template removing and nanowire releasing was achieved using NaOH (0.1 M) solution for 3 hours. Finally, the nanowires were washed with PBS solution (pH = 7.4) and centrifuged (10,000 rpm, 5 min) for three times, and finally stored in PBS solution (4 °C) for further uses.

### Fabrication and characterization of the sperm-like nanorobots

The as-prepared Au/PPy nanowires and biotin modified Fe_3_O_4_ nanoparticles were mixed in solution at a ratio of 1:1, and gently shaken at room temperature for 30 min. Due to the solid bonding between biotin and streptavidin, artificial sperm-like nanorobots were formed via self-assembly and nanorobots with one single flagellum could be mostly investigated in the experiments. The morphology and main elements of the as-prepared flagella and nanorobots were characterized by a field emission scanning electron microscope (SU-8010LA, Hitachi) equipped with an energy dispersive spectrometer.

### Magnetic actuation experiments of the sperm-like nanorobots

A custom-made triaxial Helmholtz coil system was used to actuate the nanorobotic sperms, and the details were demonstrated in our previous published papers^10,36^. To actuate the sperm-like nanorobot and test its propulsion performance, a precessing magnetic field was generated via the coils, in which the resultant field vector at the head of the nanorobot was rotating in a cone-like path. The field strength, frequency and spatial directions could be precisely tuned via a programmed current controller coupled with the coils. Locomotion of the nanorobots could be recorded in real time through a CMOS camera installed on the microscope. In our experiments, the as-prepared nanorobotic sperms were dispersed in 60% glycerin solution and transferred into a PDMS chamber at the coil center for magnetic actuation tests. In the viscous medium, the nanorobots could be considered as floating and adverse effect of the substrate surface was eliminated. For velocity measurement and locomotion direction tests, three times of experiments were conducted, and average velocity as well as standard deviations were calculated based on the experimental data.

## Supplementary Information

Supplementary Video 1.

Supplementary Video 2.

Supplementary Video 3.

Supplementary Video 4.

Supplementary Video 5.

Supplementary Video 6.

Supplementary Video 7.

Supplementary Video 8.

Supplementary Information 1.
