# Supplementary material for: Artificial flexible sperm-like nanorobot based on self-assembly and its bidirectional propulsion in precessing magnetic fields
Source: Sci Rep. 2021 Nov 5;11:21728. doi: 10.1038/s41598-021-00902-6 (PMC8571375; doi:10.1038/s41598-021-00902-6)
Supplement: Supplementary file 9 — Supplementary Information 1. [file 41598_2021_902_MOESM9_ESM.pdf]

Supporting Information for:

Artificial Flexible Sperm-like Nanorobot based on Self-assembly and Its Bidirectional Propulsion in Precessing Magnetic Fields

Nuoer Celi[a,*], De Gong[a,b,*,#], Jun Cai[a]

[a]School of Mechanical Engineering and Automation, Beihang University, Beijing, China

[b]Shen Yuan Honors College, Beihang University, Beijing, China

[*]The authors contributed equally to this work.

[#]Corresponding author, Email address: gongde@buaa.edu.cn

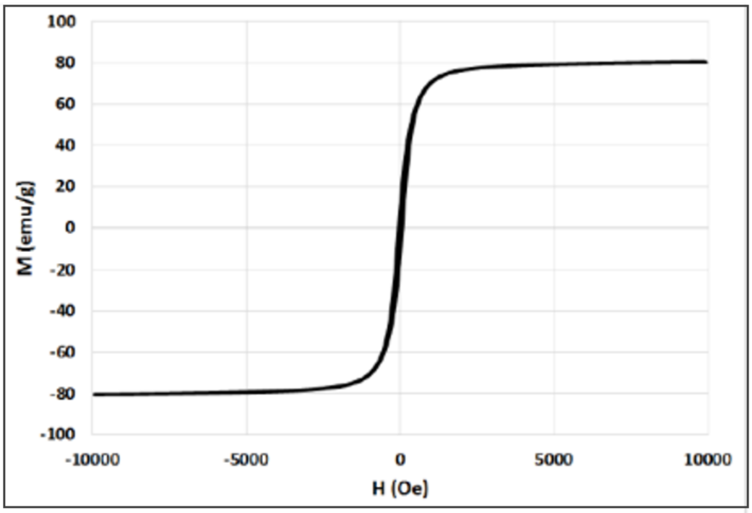

Fig. S1 VSM magnetization curves of the Fe_3O_4 heads [1].

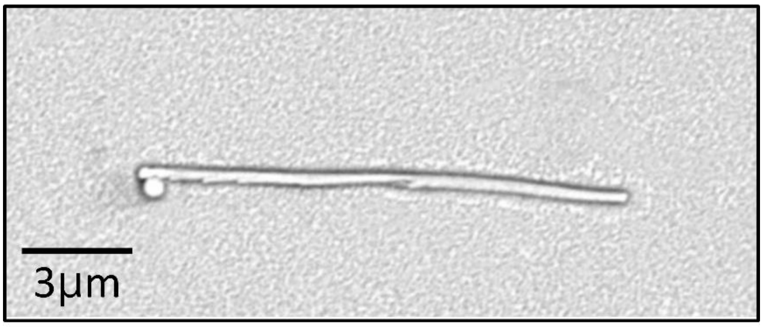

Fig. S2 SEM image of one long-tailed sperm-like nanorobot. The length of the flagellum was about 13 μm.

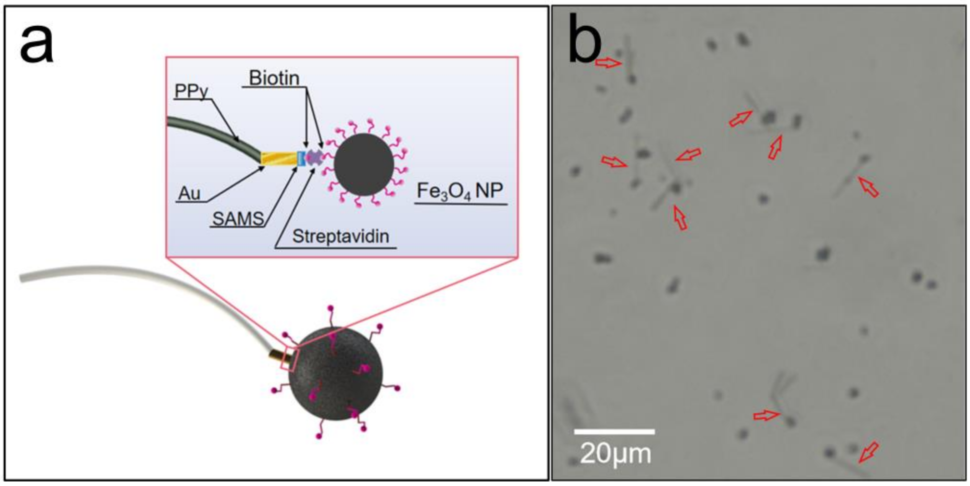

Fig. S3 (a) Structural schematics and (b) optical image of the self-assembled sperm-like nanorobot.

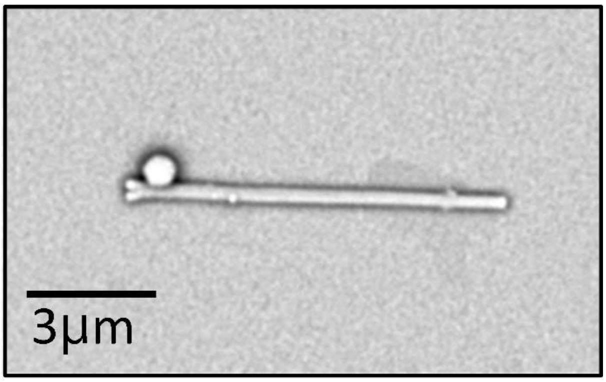

Fig. S4 SEM image of one short-tailed sperm-like nanorobot. The length of the flagellum was about 8.5 µm.

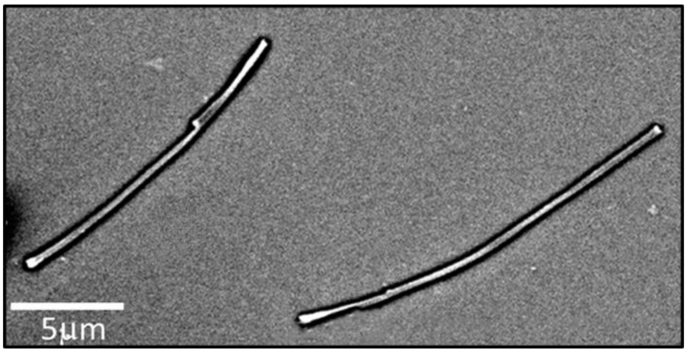

Fig. S5 SEM image of the flexible artificial flagella.

Movie 1: Flagellar oscillation investigation of a sperm-like nanorobot under externally applied precessing magnetic field. The field parameters were set as: B=100 Gs, f=2 Hz, θ=30°, and the directional type was HF-CCW.

Movie 2: Flagellar propulsion of a sperm-like nanorobot under diverse precessing magnetic fields. The field parameters were set as: B=100 Gs, f=40 Hz, θ=30°, yet the directional type and precessing angles were set to be HF-CCW (θ=10°), HF-CW (θ=10°), FF-CCW (θ=10°), FF-CW (θ=10°), HF-CCW (θ=30°), HF-CW (θ=30°) in sequence. And the corresponding bidirectional locomotion could be clearly observed. The movie was played at twice the real-time speed.

Movie 3: A short-tailed sperm-like nanorobot actuated under a given precessing magnetic field. The field parameters were set as: B=100 Gs, f=20 Hz, θ=30°, yet the directional type were set to be HF-CCW, HF-CW, FF-CCW, FF-CW in sequence. And the corresponding bidirectional locomotion could be clearly observed. The movie was played at twice the real-time speed.

Movie 4: A short-tailed sperm-like nanorobot actuated under a given precessing magnetic field. The field parameters were set as: B=100 Gs, f=30 Hz, θ=30°, yet the directional type were set to be HF-CCW, HF-CW, FF-CCW, FF-CW in sequence. And the corresponding bidirectional locomotion could be clearly observed. The movie was played at twice the real-time speed.

Movie 5: Two magnetic nanowires succeeded to turn around when the precessing axis of the actuation field was changed. The field parameters were set as: B=70 Gs, f=2 Hz, θ=30°, yet the directional type were set to be HF-CCW, HF-CW, FF-CCW, FF-CW in sequence. The magnetic nanowires used in Movie 5 and 6 were 1-link Ni-Au-PPy, which were synthesized via electrochemical deposition method according to the previous published research [2].

Movie 6: Two magnetic nanowires failed to turn around when the precessing axis of the actuation field was changed. The field parameters were set as: B=70 Gs, f=5 Hz, θ=30°, yet the directional type were set to be HF-CCW, HF-CW, FF-CCW, FF-CW in sequence.

Movie 7: A long-tailed sperm-like nanorobot actuated under a given precessing magnetic field. The field parameters were set as: B=50 Gs, f=15 Hz, θ=60°, yet the directional type were set to be HF-CW, HF-CCW, FF-CW, FF-CCW in sequence. Smooth transition and corresponding bidirectional locomotion could be clearly observed. The movie was played at twice the real-time speed.

Movie 8: A sperm-like nanorobot actuated under a given precessing magnetic field to demonstrate its flagellar flexibility. The HF-CCW field were set as: B=50 Gs, f=5 Hz, θ=10°.

References

[1] SPHERO™ Paramagnetic and Superparamagnetic Particles. http://www.Spherotech.com.

[2] B. Jang, E. Gutman, N. Stucki, B.F. Seitz, P.D. Wendel-García, T. Newton, J. Pokki, O. Ergeneman, S. Pané, Y. Or, B.J. Nelson, Undulatory Locomotion of Magnetic Multilink Nanoswimmers, Nano Lett., 15 (2015) 4829-4833, https://doi.org/10.1021/acs.nanolett.5b01981.
